# A human genotyping trial to estimate the post-feeding time from mosquito blood meals

**DOI:** 10.1371/journal.pone.0179319

**Published:** 2017-06-15

**Authors:** Yuuji Hiroshige, Masaaki Hara, Atsushi Nagai, Tomoyuki Hikitsuchi, Mitsuo Umeda, Yumi Kawajiri, Koji Nakayama, Koichi Suzuki, Aya Takada, Akira Ishii, Toshimichi Yamamoto

**Affiliations:** 1 Department of Legal Medicine and Bioethics, Graduate School of Medicine, Nagoya University, 65 Tsurumai-cho, Showa-ku, Nagoya, Japan; 2 Department of Forensic Medicine, Saitama Medical University, 38 Morohongo, Moroyama, Saitama, Japan; 3 Department of Legal Medicine, Graduate School of Medicine, Gifu University, Yanagito, Gifu, Japan; 4 Research & Development Laboratory, Dainihon Jochugiku Co., Ltd., 1-chome, Daikoku-cho, Toyonaka-shi, Osaka, Japan; 5 Department of Legal Medicine, Osaka Medical College, 2–7 Daigaku, Takatsuki, Japan; Institut Pasteur, FRANCE

## Abstract

Mosquitoes occur almost worldwide, and females of some species feed on blood from humans and other animals to support ovum maturation. In warm and hot seasons, such as the summer in Japan, fed mosquitoes are often observed at crime scenes. The current study attempted to estimate the time that elapsed since feeding from the degree of human DNA digestion in mosquito blood meals and also to identify the individual human sources of the DNA using genotyping in two species of mosquito: *Culex pipiens pallens* and *Aedes albopictus*. After stereomicroscopic observation, the extracted DNA samples were quantified using a human DNA quantification and quality control kit and were genotyped for 15 short tandem repeats using a commercial multiplexing kit. It took about 3 days for the complete digestion of a blood meal, and genotyping was possible until 2 days post-feeding. The relative peak heights of the 15 STRs and DNA concentrations were useful for estimating the post-feeding time to approximately half a day between 0 and 2 days. Furthermore, the quantitative ratios derived from STR peak heights and the quality control kit (Q129/Q41, Q305/Q41, and Q305/Q129) were reasonably effective for estimating the approximate post-feeding time after 2–3 days. We suggest that this study may be very useful for estimating the time since a mosquito fed from blood meal DNA, although further refinements are necessary to estimate the times more accurately.

## Introduction

The mosquito, one of the most common and widespread insects, belongs to the order Diptera, the suborder Nematocera, and the family Culicidae. Up to about 3,600 species in 37 genera are found worldwide [1], mainly in temperate and tropical regions. Some are vectors for diseases such as malaria, dengue fever, and chikungunya, and thus cause many deaths each year. Both male and female mosquitoes imbibe sugars in the form of flower nectar, and tree sap. However, only the females consume blood to support the maturation of ova. Aspects of feeding behavior vary among species, including the time of day (daytime, dusk, or night), hunting method (ambush or forage), location (indoors or outdoors), and the preferred host species (such as humans, dogs, pigs, cattle, horses, and birds) [2, 3].

The body of a mosquito comprises the head, thorax, and abdomen, the surfaces of which are covered with a hard-protective chitin exoskeleton. The abdomen is divided into 10 segments. Segments one to eight are each composed of a dorsal tergum and a ventral sternum that are connected by a pleurite. The remaining segments form the genitalia. When a female mosquito feeds on blood (through the proboscis and esophagus), the pleural membrane stretches to inflate the whole abdomen; blood is stored in the midgut. In contrast, when sugar is imbibed it is stored in the ventral diverticulum and transferred incrementally to the midgut for digestion [2–5].

Female mosquitoes identify and approach their targets using sensors for exhaled carbon dioxide, lactic acid in perspiration, color, and body temperature. Since the weight of the blood meal is approximately the same as that of the body [3], it seems to be impossible for them to fly far after feeding. Therefore, it is assumed that they must remain nearby for the time necessary to digest the blood to provide the nutritional resources needed for egg maturation. However, the behavior of mosquitoes after blood meals varies and depends on the species [3, 6]. Some studies have investigated animal blood meals in a zoo and revealed that the flight distances of mosquitoes after blood meals ranged from about 91.4–128.0 m, on average, among four species [7] or from 15.5 to 327.0 m at a few Sella stages among several species [8]. It was reported to take about 3 days for *Culex pipiens pallens* (*CPP*) to completely digest a blood meal at 25°C [3, 5]. It is inferred that any DNA present in such a blood meal would be degraded during digestion.

Mosquitoes, mainly *CPP* and *Aedes albopictus* (*AA*), are common outdoors and indoors in Japan from early summer (around May) to late fall (around November); in winter, they may occasionally be seen indoors. Both dead and live mosquitoes are often observed at crime scenes in Japan, especially indoors in summer. We wondered whether it is possible to identify the bitten individuals and estimate the post-feeding (PF) time using the gut contents of the mosquitoes.

Many previous studies have investigated fed mosquitoes [6–18]. Some have reported species identification using immunoprecipitation with antisera from various animals [6, 9], classical blood typing for ABO, MN, and Rh, serum protein typing for group-specific component (Gc) and haptoglobin (Hp), and red cell enzyme typing for phosphoglucomutase (PGM), acid phosphatase (AcP), and esterase D (EsD) [10, 11]. Other studies used DNA-based techniques such as identifying the host species using the *cytochrome b* and/or *cytochrome oxidase* genes in the mitochondrial DNA (mtDNA) by direct sequencing [7, 8, 12, 13] and polymerase chain reaction with the restriction fragment length polymorphisms (PCR-RFLPs) [14, 15]. Some studies have investigated human personal identification using variable number tandem repeats (VNTRs) and/or short tandem repeats (STRs) genotyping with gel electrophoresis followed by silver staining [16, 17], and two multiplex STR genotyping studies with capillary electrophoresis [18] have been published. The former also referred to the time limits of qualitative band detection [16], or the time period within which the bands could be detected macroscopically [16, 17]. In the latter study [18], the study would not be able to follow because of the ethical issues; specifically, only one donor among the co-authors was fed blood to mosquitoes, which were doubted to be infected or uninfected, more than 150 times in an uncontrolled open field. Methodologically, a less accurate quantification kit based on slot blot detection was used [16], as well as an accurate quantitative PCR method that was not appropriate for estimating DNA quality (a degree of DNA degradation) [18]. Furthermore, less accurate genotyping was performed by reducing the PCR reaction volume by half, and the number of PCR cycles was increased from 28 to 30 or 32 for the degraded DNA template not according to the manual [18]. Together, this increased the chances of “drop-in” and/or “drop-out”.

Currently, multiplex STR typing kits for more than a dozen human loci with a high discrimination power are commonly used, and the data can be digitized using computationally detected peak numbers, peak heights (PHs), peak areas, and the ratios of those data. A new commercial human DNA quantification kit that can assay the degree of DNA fragmentation to assess DNA quality has also been released. Furthermore, the availability of some mosquito species that are maintained under controlled conditions in exclusive cages has allowed procedures to be developed that keep blood-fed mosquitoes for several days, from the time of feeding until death.

The current study examined the extent to which DNA in a blood meal can be identified over PF time using multiplex STR genotyping, according to the manufacturer’s protocol. Furthermore, the time that elapsed since feeding was estimated by assaying the DNA quantity and integrity using quantitative PCR for three sizes of PCR amplicons, as well as the number and height of the signal peaks from the STR loci detected during genotyping. As this study was performed using two species of mosquitoes found both in Japan and extensively across multiple continents, it is likely that the same procedures will be applicable to other mosquito species in other countries. Therefore, a novel systematic and controllable procedure is proposed for genotyping multiple STRs and estimating the time since blood feeding more accurately. With ethical approval, it will be possible for all researchers to follow or improve this procedure.

## Materials and methods

### Mosquitoes

Two species of female mosquito, *CPP* and *AA*, were supplied by Dainihon Jochugiku Co., Ltd. (Osaka, Japan) and grown at 25°C under 12 h:12 h light: dark cycles with 3% sucrose solution as a food source. Individuals from both species were isolated in separate cages before use. They were denied access to a blood meal from the egg stage to imagoes about 6 days for *CPP* and about 5 days for *AA* after eclosion to avoid infectious diseases. Each mosquito was picked at approximately the same size: 5.5 mm for *CPP* and 4.5 mm for *AA*.

### Feeding experiments for sample preparation

Seven volunteers cooperated with the feeding experimental procedures and provided written informed consent using the form approved by the ethics committee of Saitama Medical University (approval number 765). Each female mosquito picked was placed into a transparent plastic cup (ϕ = ca. 7 cm, H = ca. 4 cm) covered with a thin net (cut from white nylon stockings) as the lid. Then, a cup containing a female mosquito was set net-side down at an appropriate place on each arm of each volunteer for an appropriate time to allow sufficient blood to be obtained to finish the meal intentionally though the net. Each fed mosquito was kept in an air-conditioned room at 25°C under a 12 h: 12 h light: dark cycle with 3% sucrose solution. Next, mosquitoes from each species that were fed from seven volunteers were sacrificed at 0, 1, 2, 3, 4, 6, 8, 12, 18, 24, 36, 48, or 72 h after feeding by placing the plastic cup containing the fed mosquito into a plastic bag filled with diethyl ether gas. Mosquitoes without blood feeding were harvested at the same time intervals and used as negative controls.

Each fed mosquito was photographed with a portable microscope (MJ-39; SATOTECH, Tokyo, Japan) for stereomicroscopic examination and quickly placed in a microcentrifuge tube containing 180 μL of ATL buffer at 4°C for DNA extraction (described below). The tubes were immediately stored at –80°C.

### DNA extraction

Prior to DNA extraction, a pipette tip was used to break up each mosquito abdomen as much as possible in thawed ATL buffer to improve lysis of the exoskeleton with proteinase K [2]. DNA was extracted using a QIAamp DNA Micro kit (Qiagen, Hilden, Germany) after modifying the manufacturer’s protocol for dried blood spots [19] by changing the elution volume (to 20 μL), number of times for elutions (to two), and elution buffer (to low TE) [20].

### DNA quantification

The extracted DNA was amplified using a KAPA Human Genomic DNA Quantification and QC Kit (Kapa Biosystems, Inc., Massachusettes, USA), in which three primer sets are included to amplify three sizes of PCR fragments (41, 129, and 305 bp) for each DNA region, according to the manufacturer’s protocol [21]. A StepOnePlus™ Real-Time PCR System (Life Technologies, Massachusetts, USA) was used to generate standard curves using routine procedures [22]. The second derivative maximum method (relative threshold cycle method) [23] was adopted to calculate the DNA concentrations (Qs: Q41, Q129, and Q305) for each amplicon size (41, 129, and 305 bp, respectively). Q-ratios (Q129/Q41, Q305/Q41, and Q305/Q129) were also calculated [21].

### Genotyping

One-microliter of extracted DNA was amplified in 28 PCR cycles using an AmpFℓSTR^®^ Identifiler^®^ Plus PCR Amplification Kit (IDPlus) (Life Technologies, Massachusetts, USA) for 15 STR loci and the *Amelogenin* locus (for gender determination), for a total of 16 DNA markers. One-microliter of each PCR product was capillary electrophoresed on an Applied Biosystems 3130xl Genetic Analyzer (Life Technologies), and the detected peaks were genotyped automatically using GeneMapper*ID* software ver. 3.2.1 (Life Technologies) with 50 RFU as the fixed threshold for peak detection. Most procedures were performed following the manufacturer’s protocol [24], except for the amount of template DNA because the 0.5 ng of template recommended by the protocol was not extracted from all the fed blood samples, especially after 36 h.

### Statistical analysis

Data are presented as means ± standard deviations (SDs). Student’s *t*-tests were performed using Microsoft Excel 2013 (Redmond, WA, USA). Graphs were made using Microsoft Excel 2013 and JMP Pro 11 (SAS Institute Japan, Tokyo, Japan).

## Results

### Stereomicroscopic examination

**Fig 1:**
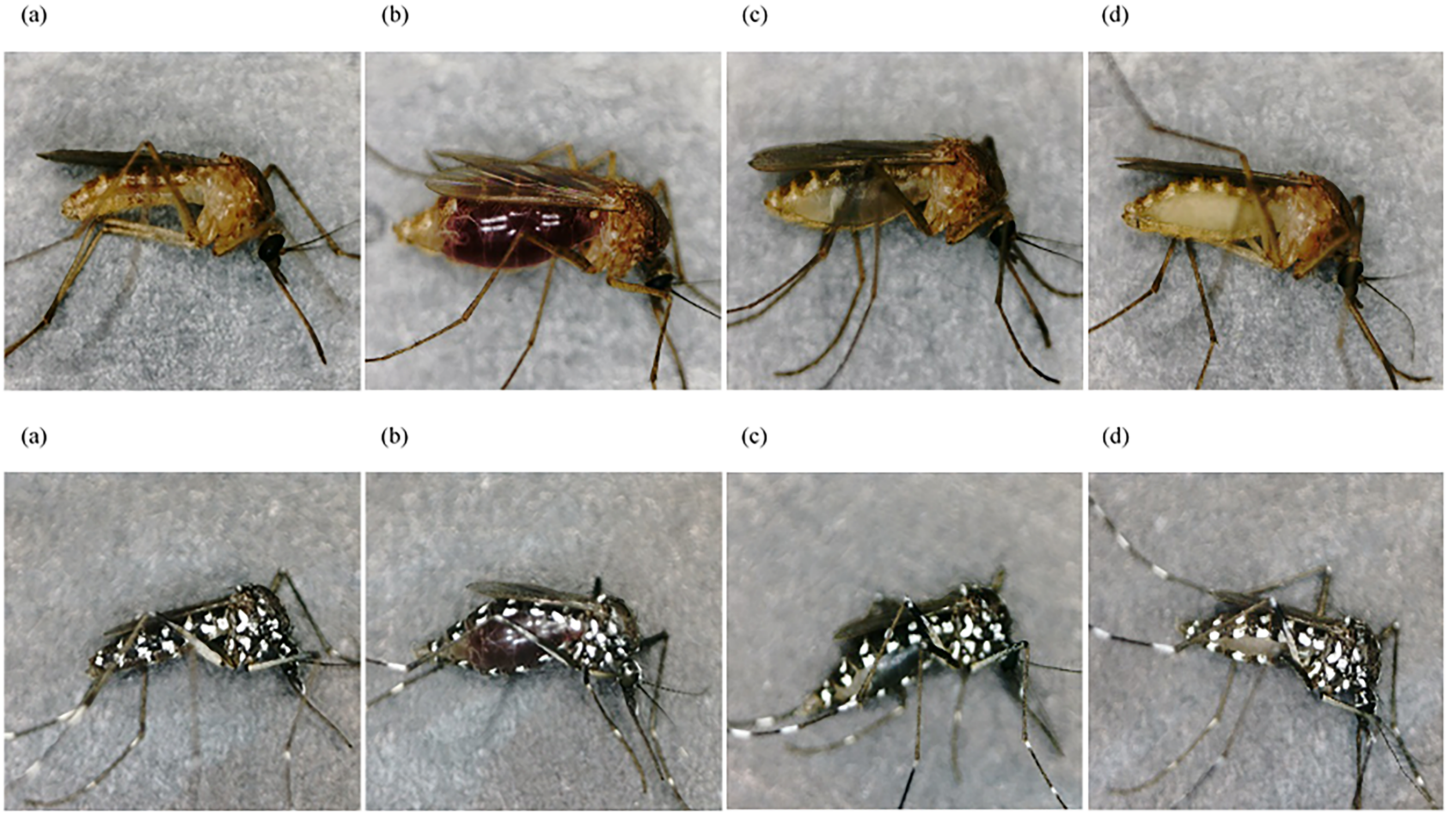
Stereomicroscopic images of both mosquito species at each time point. Upper, *Culex pipiens pallens* (*CPP*); lower, *Aedes albopictus* (*AA*). (a) Unfed, (b) 0 h, (c) 24 h, and (d) 72 h post-feeding (PF) (× ca. 20).

**Fig 2:**
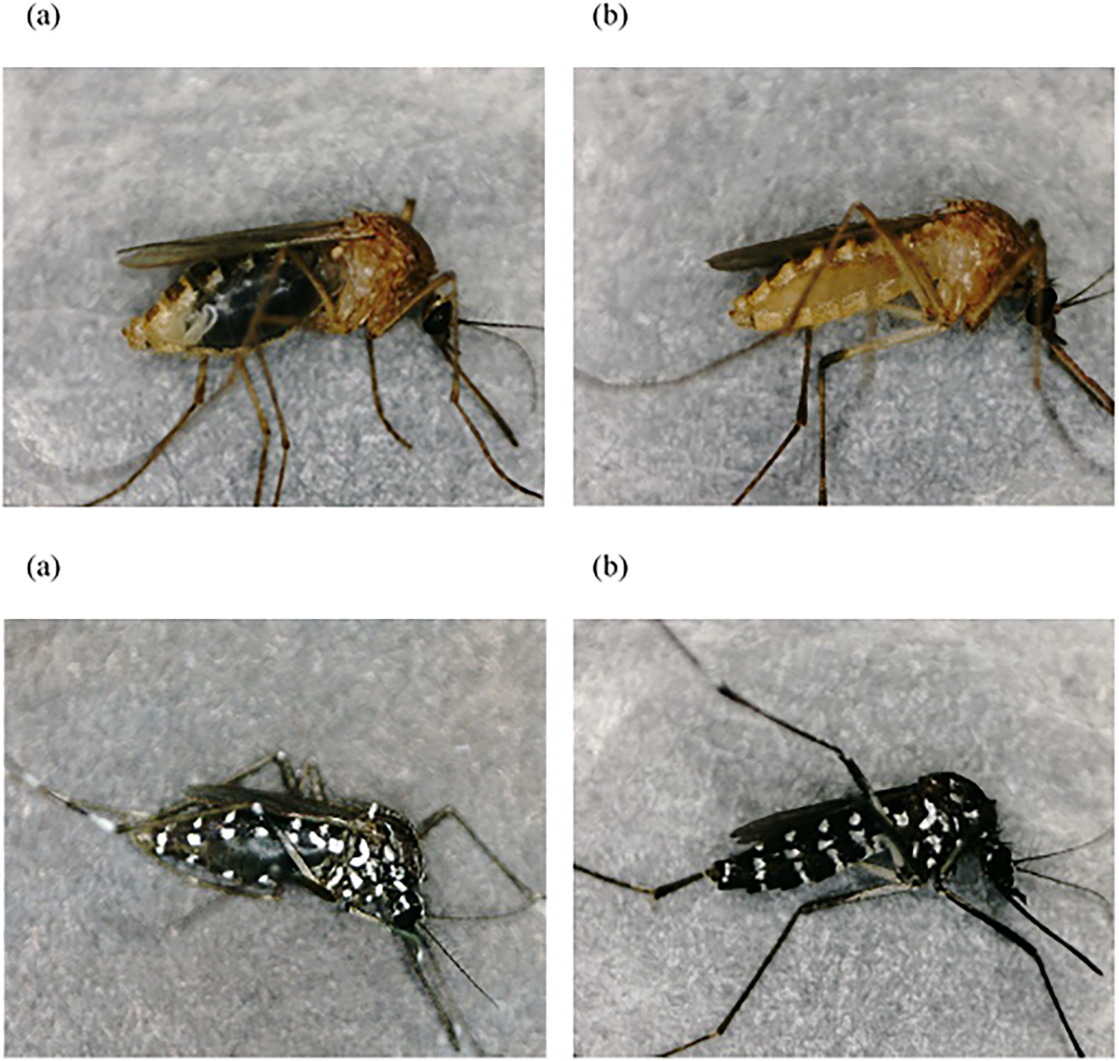
Stereomicroscopic images of both mosquito species without/with blood release at each time point. Upper, *CPP*; lower, *AA*. (a) Without and (b) with release of the blood meal at 6 h post-feeding (PF) for *CPP* and 3 h PF for *AA* (× ca. 20).

Stereomicroscopic examinations were performed using photographs of mosquitos taken just after sacrifice at each timepoint after feeding. Representative images showing changes in the color of the abdomen and the morphological alterations caused by blood digestion and ovum maturation at 0, 24, and 72 h after feeding are shown, together with an unfed negative control, in Fig 1. The abdomen color varied from red to white as the blood meals were digested and the ovaries matured. Although it was confirmed macroscopically that all the mosquitoes had taken in a significant blood meal, several of the fed mosquitoes appeared similar to unfed controls upon stereomicroscopic examination immediately after sacrifice. This was observed at earlier times PF, especially before 12 h, as shown in Fig 2. They had presumably released the blood meal at some point without our noticing. While these samples were not included in the feeding-time estimation using DNA quantification and genotyping, some of them could be genotyped (see S1 Table).

### DNA quantification

**Fig 3:**
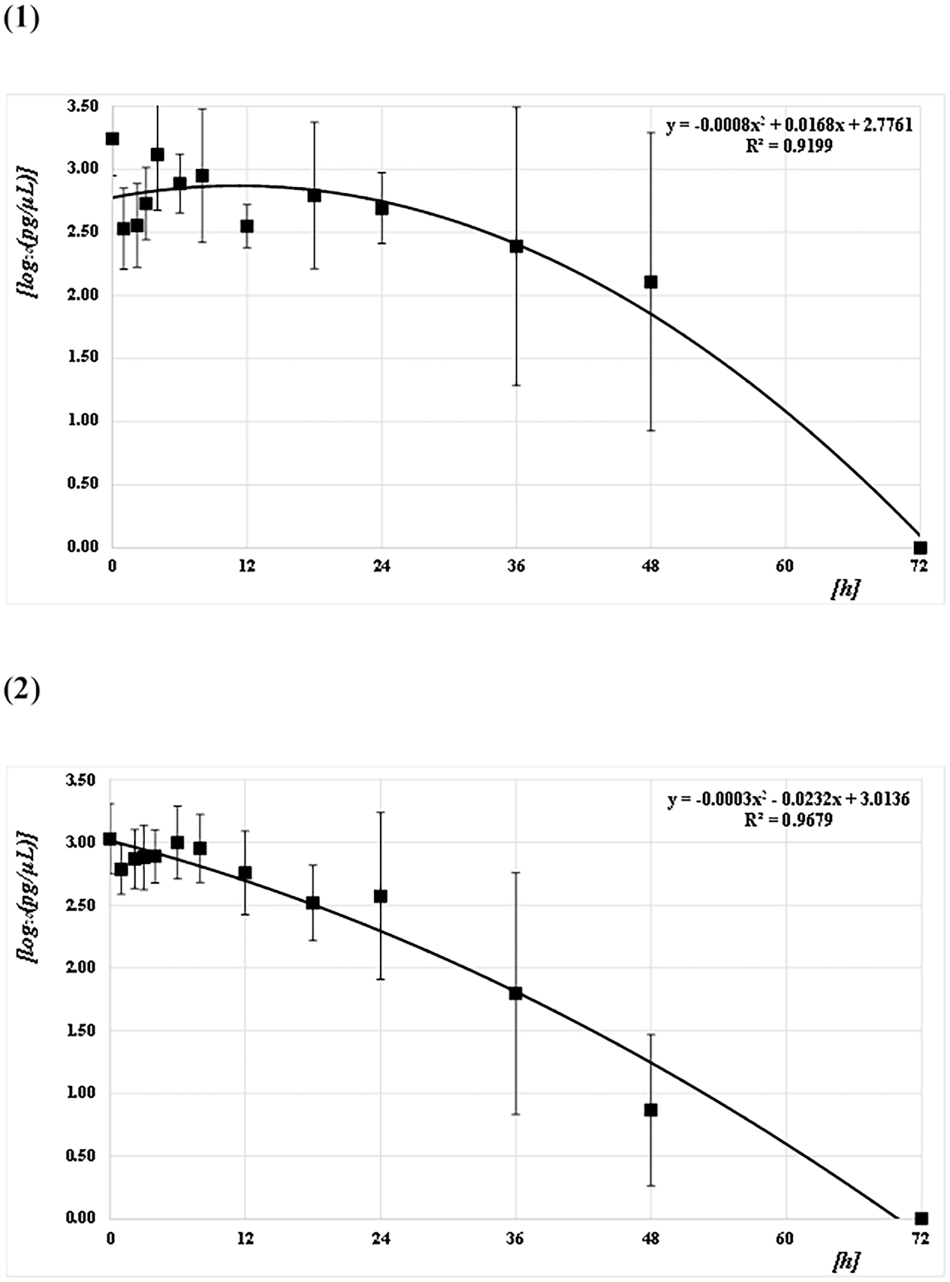
Correlation between DNA concentrations (pg/μL) and PF times (h) at Q41. (1) *CPP* and (2) *AA*. The common logarithmic values for the means and SDs of the DNA concentration at each PF time (h) are plotted as filled squares. Each quadratic regression curve for the mean plots is represented.

**Fig 4:**
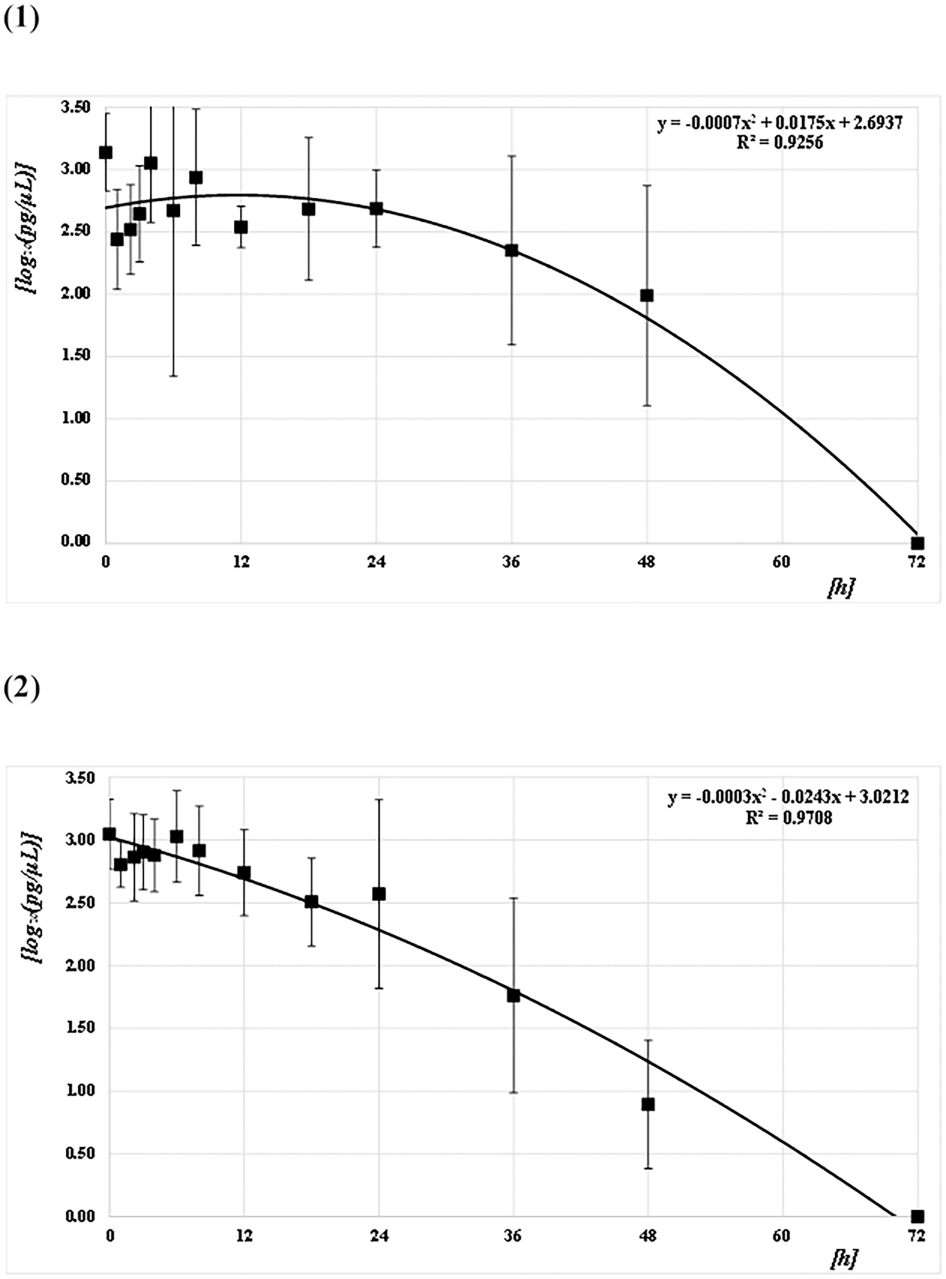
Correlation between DNA concentrations (pg/μL) and PF time (h) at Q129. (1) *CPP* and (2) *AA*. The common logarithmic values for the means and SDs of the DNA concentration at each PF time (h) are plotted as filled squares. Each quadratic regression curve for the mean plots is represented.

**Fig 5:**
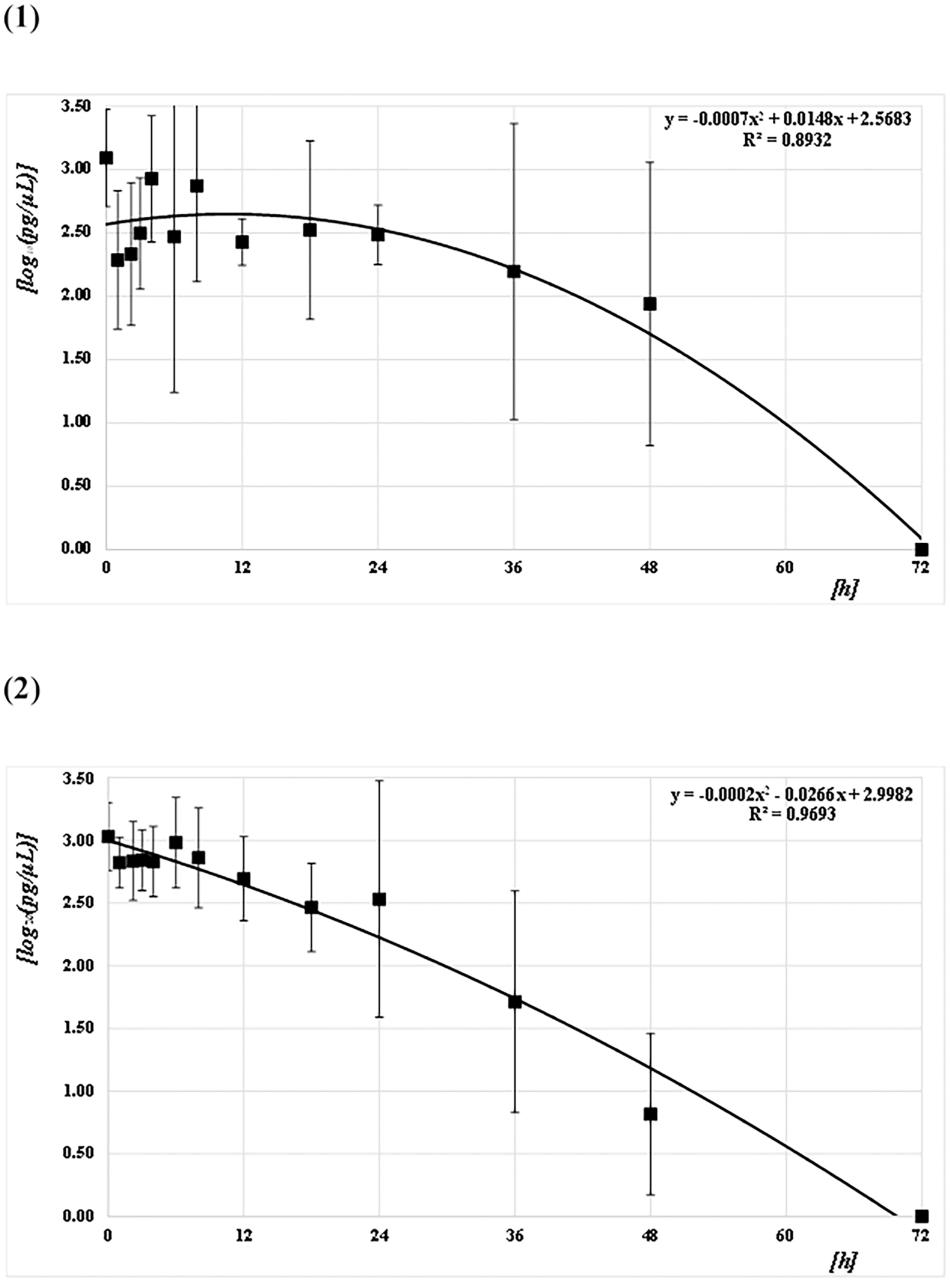
Correlation between DNA concentrations (pg/μL) and PF time (h) at Q305. (1) *CPP* and (2) *AA*. The common logarithmic values for the means and SDs of the DNA concentration at each PF time (h) are plotted as filled squares. Each quadratic regression curve for the mean plots is represented.

The concentrations of the Q41, Q129, and Q305 DNA samples from both mosquito species (*CPP* and *AA*) at each PF time point were quantified and converted to common logarithms, as shown in Figs 3–5 and S1 Table. The mean logarithmic DNA concentrations just after feeding (at 0 h) for *CPP* at Q41, Q129, and Q305 were 3.24 (1,750 pg/μL), 3.14 (1,370 pg/μL), and 3.09 (1,230 pg/μL), respectively. The amounts decreased gradually over time, but varied widely. At 72 h after feeding, DNA was undetectable in most of the samples. Regression curves were obtained from the correlation between the logarithmic DNA concentrations and PF time at Q41, Q129, and Q305, with coefficients of determination (R^2^) of 0.92, 0.93, and 0.89, respectively (Figs 3–5). The mean logarithmic DNA concentrations just after feeding for *AA* at Q41, Q129, and Q305 were 3.03 (1,070 pg/μL), 3.05 (1,110 pg/μL), and 3.03 (1,070 pg/μL), respectively. These values also decreased gradually but varied less than did samples from *CPP*. At 72 h after feeding, DNA was undetectable in most samples, as with *CPP*. Regression curves were obtained from the correlation between the logarithmic DNA concentrations and PF time for all three Qs, with R^2^ values of 0.97 (see Figs 3–5). All R^2^ values for *AA* were higher than those for *CPP*. Human DNA was not detectable in negative control samples from either species.

*T*-tests were performed to assess the pairwise statistical differences in the DNA concentrations at Q41, Q129, and Q305 among PF times; the results are summarized on Table A in S2 Table. The DNA concentrations at all amplicon sizes tended to differ significantly before and after 18 h for *AA*, but not for *CPP*.

**Fig 6:**
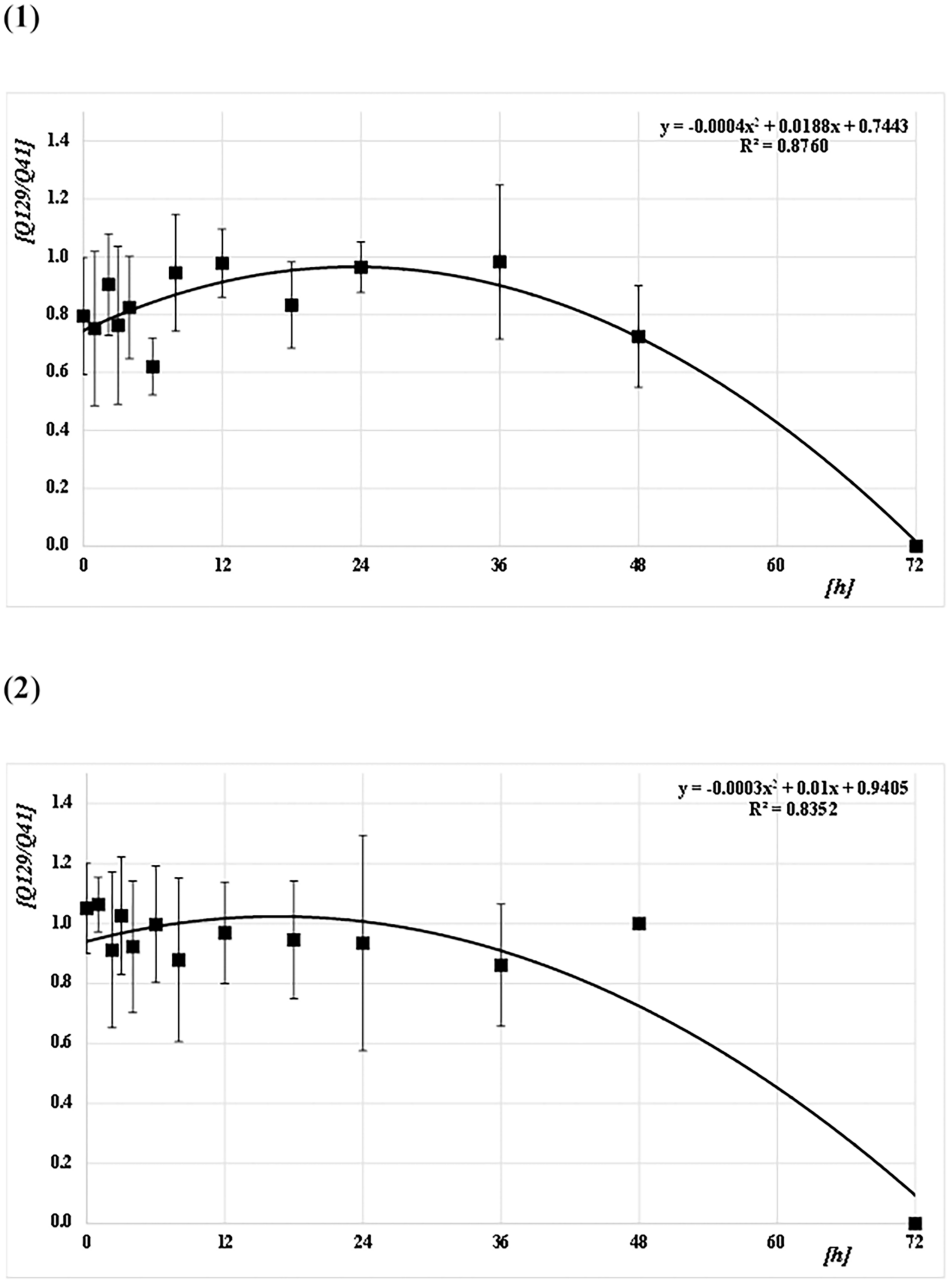
Correlation between Q-ratios and PF time (h) at Q129/Q41. (1) *CPP* and (2) *AA*. The means and SDs of the Q-ratio (Q129/Q41) at each PF time (h) are plotted as filled squares. Each quadratic regression curve for the mean plots is represented.

**Fig 7:**
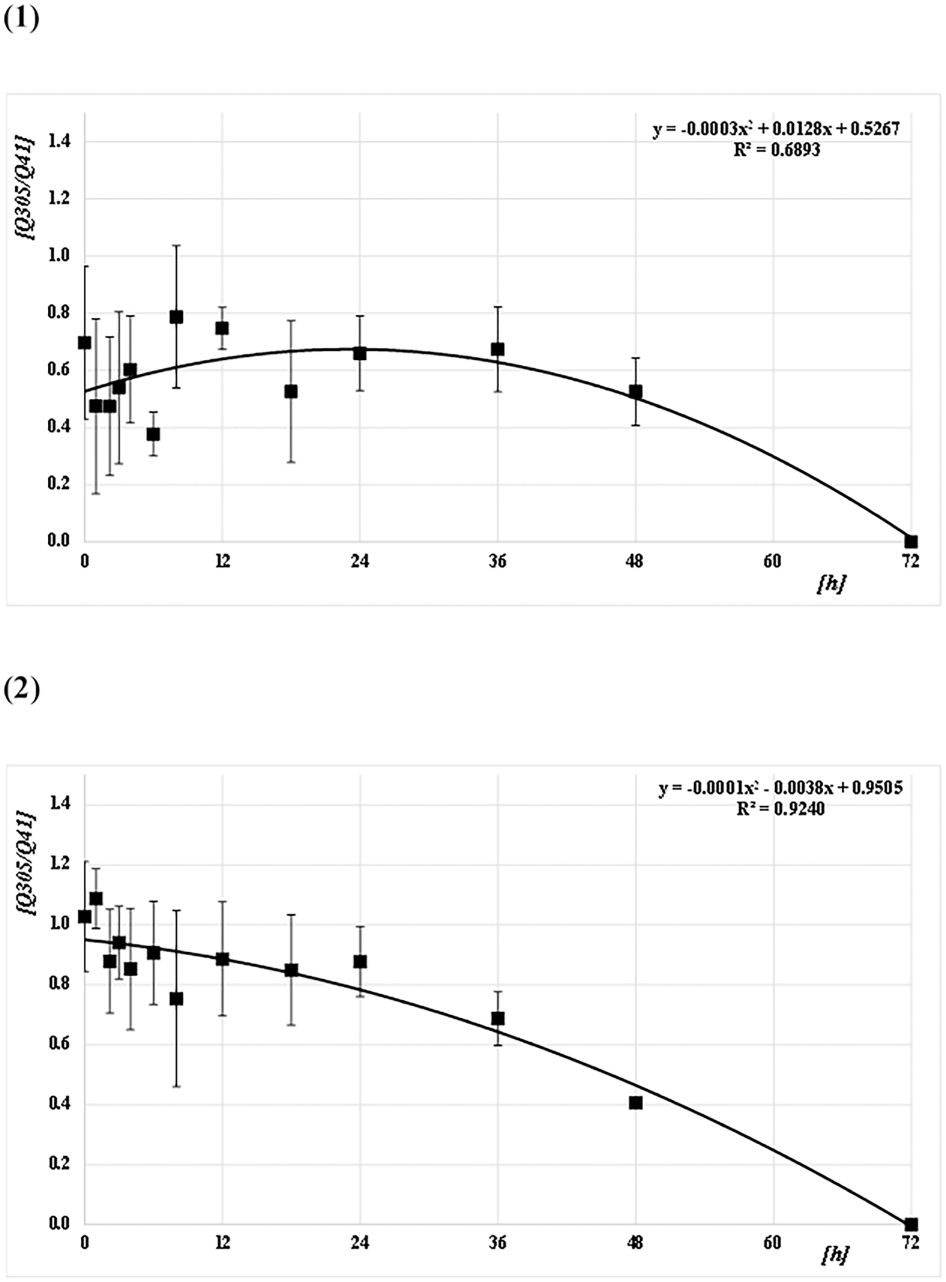
Correlation between Q-ratios and PF time (h) at Q305/Q41. (1) *CPP* and (2) *AA*. The means and SDs of the Q-ratio (Q305/Q41) at each PF time (h) are plotted as filled squares. Each quadratic regression curve for the mean plots is represented.

**Fig 8:**
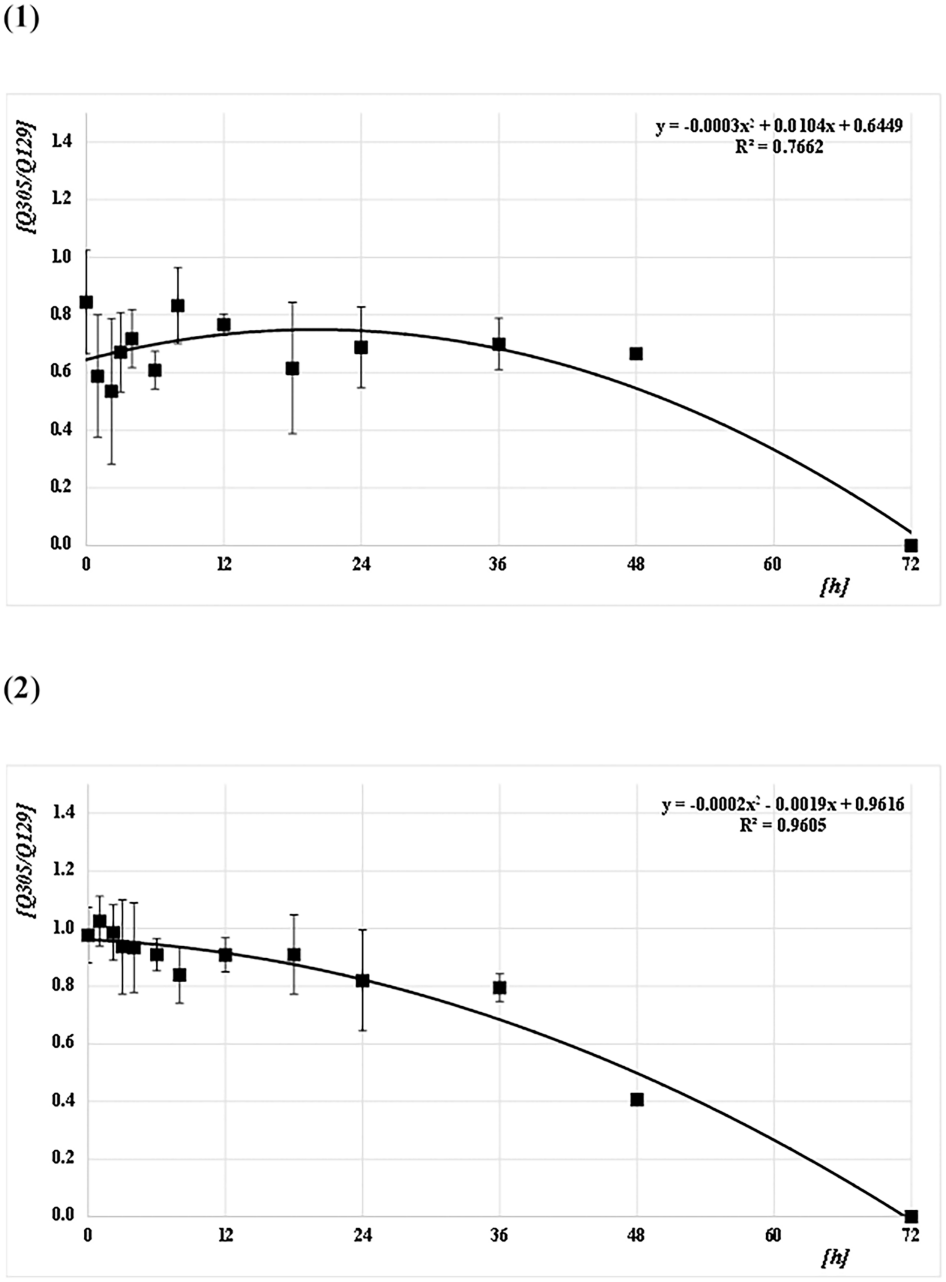
Correlation between Q-ratios and PF time (h) at Q305/Q129. (1) *CPP* and (2) *AA*. The means and SDs of the Q-ratio (Q305/Q129) at each PF time (h) are plotted as filled squares. Each quadratic regression curve for the mean plots is represented.

The Q-ratios (Q129/Q41, Q305/Q41, and Q305/Q129) were calculated from the DNA concentrations for each amplicon size (41 bp [Q41], 129 bp [Q129], and 305 bp [Q305]) for both species (*CPP* and *AA*) at each PF time (Figs 6–8 and S1 Table). For *CPP*, the mean Q129/Q41 was almost constant (about 1.0) from 0 to 48 h PF, whereas Q305/Q41 and Q305/Q129 were about 0.7. For *AA*, the mean Q129/Q41 was almost constant (about 1.0) from 0 to 48 h PF, as in *CPP*. However, Q305/Q41 and Q305/Q129 decreased gradually from about 1.0 at 0 h to 0.7 at 36 h PF and from about 1.0 at 0 h to 0.8 at 36 h, respectively. Both ratios reached about 0.4 at 48 h. At 72 h after feeding, no ratio could be calculated because most values were below the level of detection in both species. Based on Q-ratios of 0 at 72 h PF, regression curves were obtained from the correlation between Q-ratios and PF time at Q129/Q41, Q305/Q41, and Q305/Q129, with values for R^2^ of 0.88, 0.69, and 0.77 for *CPP*, and 0.84, 0.92, and 0.96 for *AA*, respectively (Figs 6–8). Generally, the mean ratios for *CPP* between 0 and 6 h PF fluctuated, whereas those for *AA* were comparatively stable.

*T*-tests were performed to assess the pairwise statistical differences between the Q-ratios at Q129/Q41, Q305/Q41, and Q305/Q129 among PF times. The results are shown on Table B in S2 Table. There were few significant differences between the Q-ratios at all amplicon sizes and all PF times in both species.

### Genotyping

Each sample was PCR-amplified using IDPlus and 1 μL of DNA extract in 28 cycles. All the alleles detected originated the from volunteers’ genomic DNA, as determined by comparisons with samples extracted from buccal swabs. No alleles were detected from the unfed negative control samples from either mosquito species.

**Fig 9:**
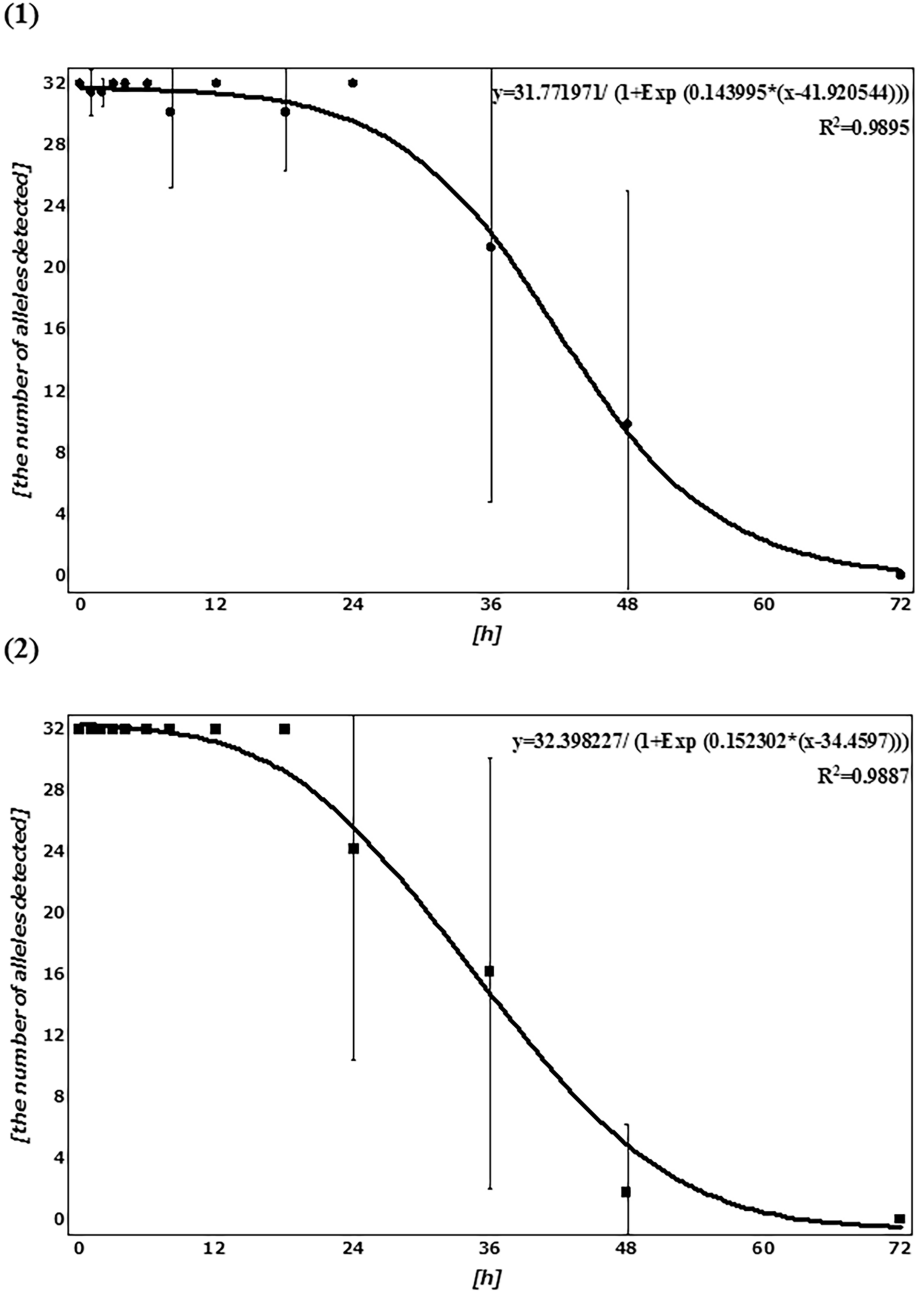
Correlation between the number of alleles detected and PF time (h). (1) *CPP* and (2) *AA*. The means and SDs of the number of alleles detected at each PF time (h) are plotted as filled circles. Each sigmoid regression curve for those mean plots is represented.

The mean number of alleles detected for each sample at each PF time is shown in Fig 9 and S1 Table. Homozygotes were presumed to generate a single peak of twice the heterozygote peak height (PH) for a single allele. On average, most human alleles present (32 alleles) were detected in *CPP* samples from 0 to 24 h PF. Then, the allele number decreased gradually over time; several alleles were detectable until 48 h and none were detected at 72 h PF. In contrast, on average, most alleles could be detected from 0 to 18 h PF in *AA* samples. Subsequently, the number decreased gradually and no alleles were detected at 72 h, as with *CPP*. Sigmoid regression curves were obtained from the correlation between the mean number of alleles detected and PF time for *CPP* and *AA*, with an R^2^ of about 0.99 for both species (Fig 9).

*T*-tests were performed to assess the pairwise statistical differences between the number of alleles detected among PF times. The results are summarized in Table B-C in S2 Table. The number of alleles detected tended to differ significantly before and after 36 h for *AA*, but not for *CPP*.

**Fig 10:**
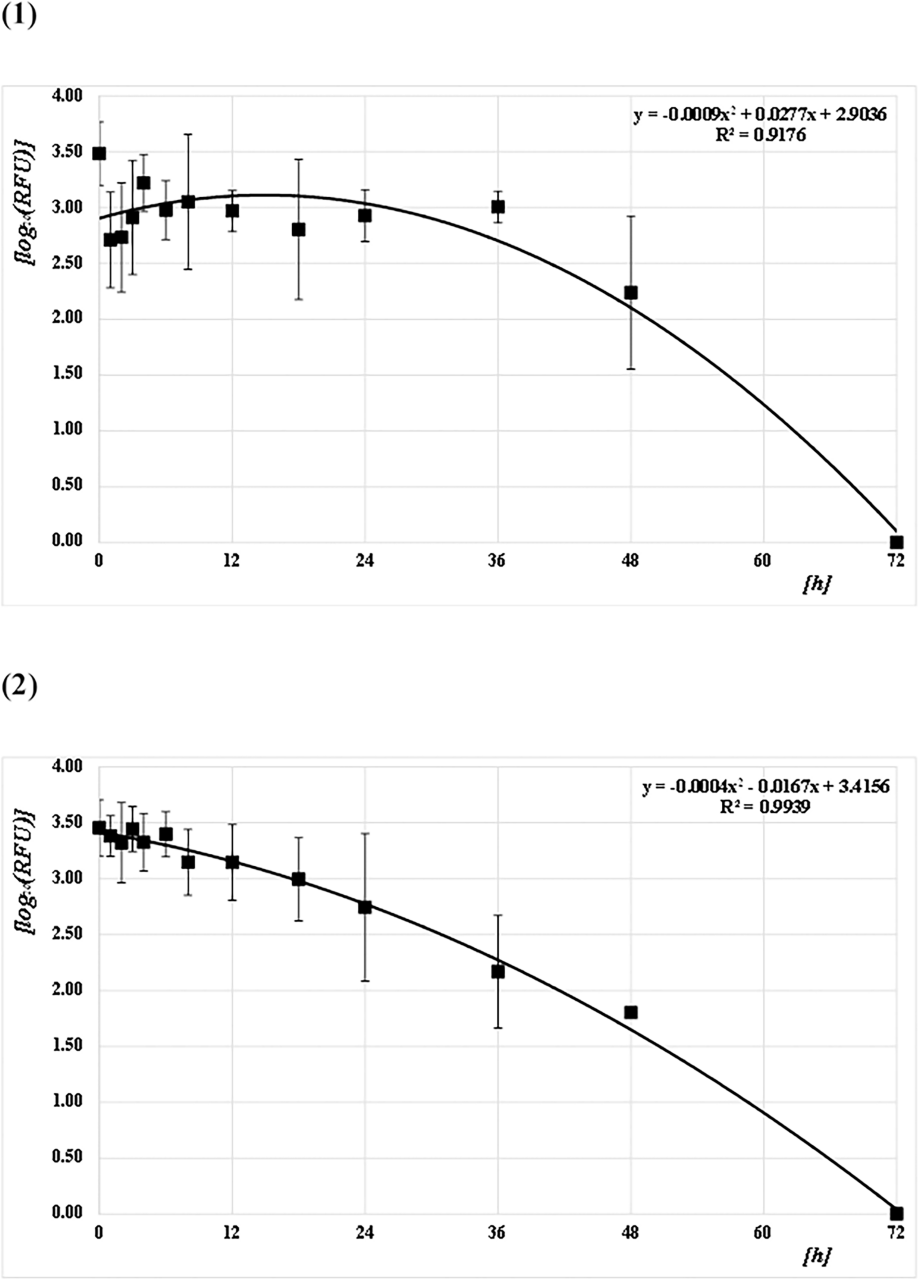
Correlation between the mean peak heights (PHs; RFU) of the detected alleles and PF time (h). (1) *CPP* and (2) *AA*. The common logarithmic values for the means and SDs of the average PHs (RFU) of the alleles detected at each PF time (h) are plotted as filled squares. Each quadratic regression curve for those mean plots is represented.

The mean PHs were calculated as the sum of the PHs for the alleles detected in each sample divided by the number of alleles detected. The PHs were calculated as half the observed height in cases of homozygous alleles. The mean PHs were converted to common logs for each PF time, as shown in Fig 10 and S1 Table. The log mean for PH just after feeding in *CPP* was 3.48 (3,600 RFU). The log averages decreased gradually, but varied widely. No alleles were detected 72 h PF, and the mean PH was 0. A regression curve was calculated from the correlation between the log mean PH and PF time, with an R^2^ of 0.92 (Fig 10). For *AA*, the log mean PH was 3.45 (3,300 RFU) at 0 h. Subsequently, the log average decreased gradually and was 0 at 72 h PF, as in *CPP*. If the log average at 72 h was 0, the correlation between log mean PH and PF time generated a regression curve with an R^2^ value of 0.99 for *AA* (Fig 10).

*T*-tests were performed to assess the pairwise statistical differences in PHs among PF times. The results are summarized in Table B-C in S2 Table. The number of alleles detected tended to differ significantly between 8 and 12 h for *AA*, but not for *CPP*.

**Fig 11:**
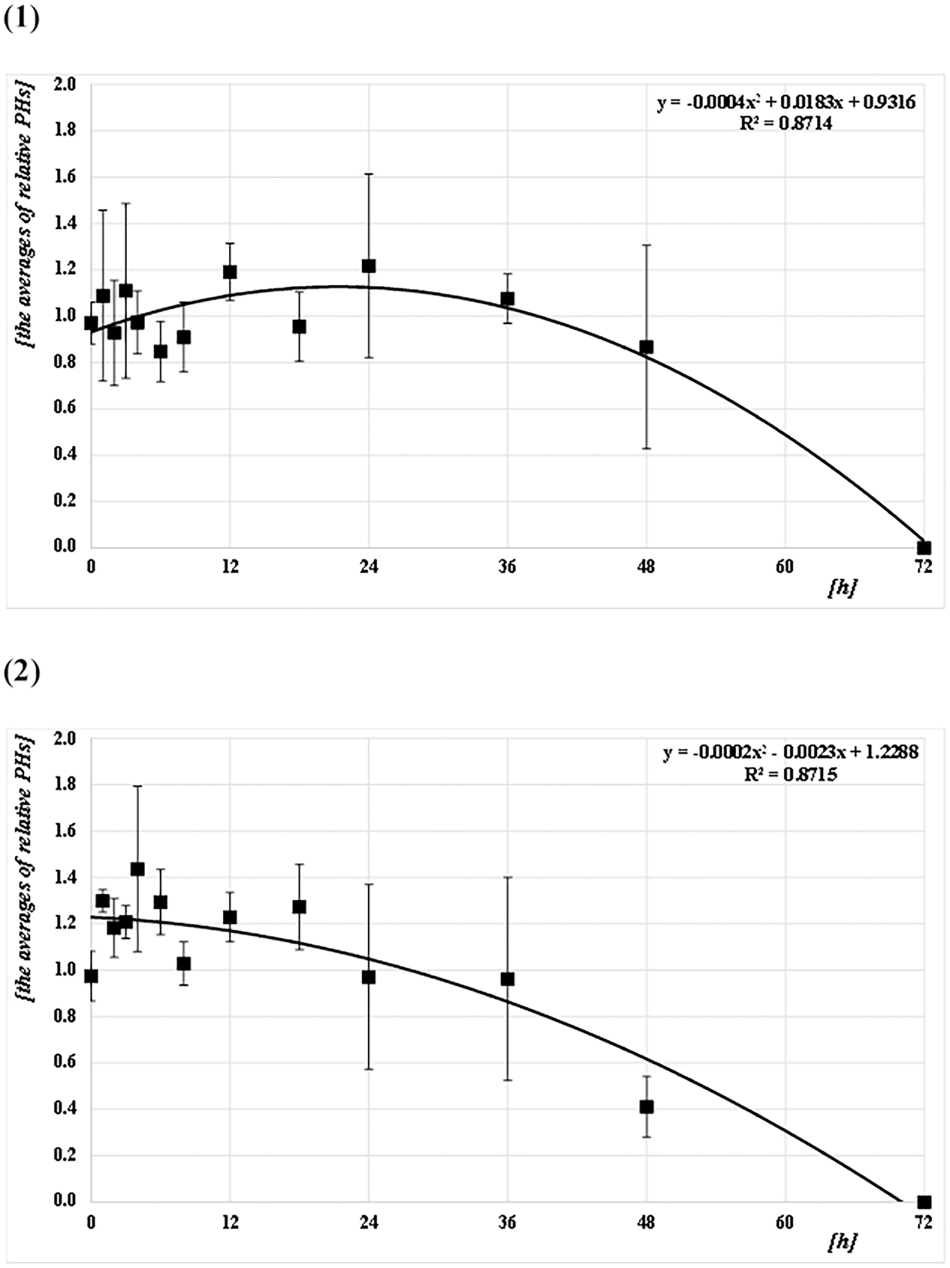
Correlation between the mean relative peak heights (PHs) of the alleles detected and PF time (h). (1) *CPP* and (2) *AA*. The common logarithmic values for the means and SDs of the average relative PHs (RFU) of the alleles detected at each PF time (h) are plotted as filled squares. Each quadratic regression curve for those mean plots is represented.

The relative PHs were calculated as follows: assuming the PH of allele X for each DNA sample was 1.0, the ratios of the PHs for all other alleles detected in the DNA sample were calculated relative to the PH of allele X. The means of the relative PHs are shown in Fig 11 and S1 Table. For *CPP*, the mean of the relative PHs was almost constant (about 1.0) from 0 to 48 h PF. For *AA*, the mean of the relative PHs also remained almost constant (about 1.0) from 0 to 36 h PF; however, the mean gradually decreased to about 0.4 by 48 h. At 72 h after feeding, relative PHs could not be calculated as no alleles were detected in either species.

*T*-tests were performed to assess the pairwise statistical differences between the mean relative PHs among PF times. The results are shown in Table B-C in S2 Table. There were few significant differences between all mean relative PHs and all PF times in both species.

## Discussion

As mentioned in the Introduction, many previous studies have investigated fed mosquito [6–18] including those using DNA-based techniques [7, 8, 12–18]. However, only a few of these [16–18] used personal identification to approximate the time after blood meals, but including some important issues as mentioned above. Current DNA profiling techniques have improved significantly by using multiplex amplification and typing systems with multi-colored fluorescent primers, and have a much higher probability for personal identification. Real-time PCR can also quantify DNA more accurately than the old-fashioned techniques used more than 10 years ago [16, 17]. Recently, although a study [18] was published using such techniques, the study is poor because only one volunteer, to whom mosquitoes were naturally attracted, was fed blood to mosquitoes in an open field where was poorly controlled, without any approval from an ethics committee. In contrast, the current study is novel and valuable because it used systematic procedures and novel techniques to perform personal identification and DNA quantification. Some quantitative values were obtained such as the DNA concentrations of multi-sized amplicons and peak heights, under well-controlled conditions (temperature and time after BF etc.) and with ethical approval. Although some novel observations were made, this exploratory study identified several problematic areas that still need to be addressed.

One unavoidable issue was the unnoticed and sudden loss of most of a blood meal, especially in *CPP*, during the earlier PF periods. This was rarer in *AA* mosquitos, leading to comparatively reliable DNA extraction results. We confirmed macroscopically that each mosquito had taken in a significant blood meal volume, that some fed mosquitoes, especially *CPP*, released blood meals slightly but not almost all while keeping until sacrificing, and that the abdomen of each fed mosquitoes was almost full with the blood meal just before sacrifice. However, some DNA extractions prior to 12 h PF yielded no quantifiable DNA, especially in *CPP*. Microscopic photographs revealed that these mosquitos had almost no blood in their abdomens. There are two possible reasons for the unnoticed disappearance of the blood meal. (1) Despite careful observation, we could have missed seeing the unpredicted release of the blood meal. We examined white gauze patches from each mosquito chamber, but did not examine the inside of each plastic cup and so may have missed any blood released there. (2) The sudden, involuntary, incontinent loss of the blood meal during sacrifice with diethyl ether may have occurred in some mosquitoes. Microscopic photographs were taken immediately after sacrifice and, because such blood losses were not expected, the inside of each plastic bag ether chamber was not observed carefully.

We believe that the latter explanation is more likely than the former for several reasons. First, it may be easier for *CPP* to release their blood meals upon sudden stimulation because their abdomen membranes are softer than those of *AA*. Therefore, this phenomenon was observed more frequently in *CPP* than in *AA*. Second, only trace amounts of DNA could be extracted from one out of seven *CPP* samples at 0 h PF. This suggests that the phenomenon was associated with sacrifice rather than the PF holding time. Third, this phenomenon was not observed frequently after 12 h PF. The more mature and larger the ovaries become over time, the more they displace the gut contents in the abdomen. In addition, the more a blood meal is digested, the more coagulated it becomes. Therefore, blood meal loss may occur easily until 12 h PF. Accordingly, the sacrifice procedure for fed mosquitoes should be modified to one that allows confirmation that such sudden blood release has been avoided.

To estimate more accurately the PF time, two main issues should be resolved. First, the number of data points should be increased to reduce the effect of variation of the sacrifice method, which has been improved. Second, more accurate methods are needed to quantify the small amounts of the slightly degraded DNA that are generated during the first 24 h of digestion, as well as the highly degraded small amounts of DNA that remain at 48 h PF. For example, a quantitative method with longer amplicons could address the former problem, and the development of an instrument with a higher sensitivity for very small amounts of DNA could solve the latter issue. Alternatively, more accurate estimates could be obtained by varying the extraction parameters and/or employing other commercially available genotyping kits.

In spite of the abovementioned issues, some reliable results were obtained in the current study, especially from *AA*. As shown in the summary for *AA* in Table 1, we suggest that the time after feeding could be estimated at approximately 12-h intervals, such as less than 12, around 12, between 12 and 24, around 24, between 24 and 36, around 36, between 36 and 48, and more than 48 h PF. When an approximate PF time is estimated from a set of values for a mosquito, each higher value would also need to be considered. For example, consider one sample in which the logarithmic mean DNA concentration at Q41 was about 2.9 (800 ng/μL), the Q129/Q41 ratio was about 1.0, the Q305/Q41 and Q305/Q129 ratios were about 0.9, the mean number of alleles detected was 32/32, the mean PH was about 3.4 (2,500 RFU), and the relative PH was about 1.3. In a second sample, the logarithmic mean DNA concentrations at Q41 and Q129 were each about 2.6 (400 ng/μL), Q305 was about 2.5 (320 ng/μL), Q129/Q41 was about 1.0, Q305/Q41 and Q305/Q129 were about 0.8, the mean number of alleles detected was 32/32, the mean PH was about 3.1 (1,260 RFU), and the relative PH was about 1.2. Using these criteria, the first of these two samples could be estimated to come from a blood meal less than 12 h PF (at 6 h PF) and the second sample from between 12 and 24 h (at 18 h PF). Approximately half of all samples from *AA* could satisfy the criteria shown in Table 1. Many of the remaining samples deviated from the standards in only one parameter. Therefore, when the PF time is estimated, the set of values for each mosquito needs to be interpreted in a comprehensive manner. Although we were unable to obtain sufficient data from *CPP*, similar results to those from *AA* would resolve the issues mentioned above.

**Table 1:** Summary of the values obtained from each regression curve for each PF time in *AA*.

Log10 DNA conc. (log10 [pg/μL])			Q-ratios			The number of alleles detected	Log10 average PH^a^	relative PH	PF time (h)
Q41	Q129	Q305	Q129 /Q41	Q305 /Q41	Q305 /Q129				
3.0	3.0	3.0	0.9	1.0	1.0	32/32	3.4	1.2	0
2.7	2.7	2.7	1.0	0.9	0.9	31/32	3.2	1.2	12
2.5	2.5	2.5	1.0	0.8	0.9	30/32	3.0	1.1	18
2.3	2.3	2.2	1.0	0.8	0.8	27/32	2.8	1.1	24
1.8	1.8	1.8	0.9	0.7	0.6	14/32	2.3	0.9	36
1.2	1.2	1.2	0.7	0.5	0.4	4/32	1.7	0.7	48
ND^b^			NC^c^			0/32	ND	NC	72

When an approximate PF time was estimated from a set of values for a mosquito, each higher value must also be considered. PF, post-feeding

^a^Peak height.

^b^Not detectable.

^c^Not calculable.

The current study successfully developed a basic estimation of PF time, especially for *AA*. More accurate estimations should be possible after improvements in the quantitation method, resolution of the blood-meal release issue, and increasing the number of samples.

## Supporting information

S1 Table Summary of all of the mean and SD data used to generate Figs 3–11. (XLSX)

S2 Table Pairwise differences among post-feeding (PF) times (h) with P-values generated using *t*-tests. The values in red were significantly different (P<0.05).
Table A: The DNA concentrations at Q41, Q129, and Q305 for *CPP* and *AA* are shown in the upper and lower tables, respectively.
Table B: The Q-ratios at Q129/Q41, Q305/Q41, and Q305/Q129 for *CPP* and *AA* are shown in the upper and lower tables, respectively.
Table C: The number of alleles detected, the average and relative peak heights (PHs) of the alleles detected for *CPP* and *AA* are shown in the upper and lower tables, respectively.
(XLSX)

## References

[1] Harbach RE, Kitching IJ. The phylogeny of Anophelinae revisited: inferences about the origin and classification of *Anopheles* (Diptera: Culicidae). Zoologica Scripta. 2015; 45(1): 34‒47. doi: 10.1111/zsc.12137

[2] Kamimura K. Mosquitoes and the control of mosquito-borne diseases. The society of Urban Pest Management. 2004; 26 (1): 25–54. [in Japanese]

[3] Sasa M, Kurihara T, Kamimura K. Science of the Mosquito (Kanokagaku). Tokyo: Zukannohokuryuukan Press; 1976. [in Japanese]

[4] Arai M. Infectious Disease-Mediated Mosquito in Japan (Nihonniokerukansensyou -baikaikai). Modern Media. 2012; 58(6): 199–203. [in Japanese]

[5] Detinova TS. Age-grouping methods in Diptera of medical importance with special reference to some vectors of malaria. World Health Organization Monograph Series. 1962; 47: 54–55. 13885800

[6] John DE, William LB. Flight capacity of blood-engorged mosquitoes. Mosquito News. 1969; 29(3): 386–392.

[7] Jacob AG, Mark AD, Ben H, Bruce VH. Analysis of post-blood meal flight distances in mosquitoes utilizing zoo animal blood meals. J Vector Ecol. 2012; 37(1): 83–89. doi: 10.1111/j.1948-7134.2012.00203.x 22548540 10.1111/j.1948-7134.2012.00203.x PMC3342775

[8] Tuten HC, Bridges WC, Paul KS, Adler PH. Blood-feeding ecology of mosquitoes in zoos. Med Vet Entomol. 2012; 26(4): 407–416. doi: 10.1111/j.1365-2915.2012.01012.x 22390304 10.1111/j.1365-2915.2012.01012.x

[9] Gower A.K.O. The Rate of Digestion of Human Blood by Certain Species of Mosquitoes. Aust J Biol Sci. 1955; 9(1): 125–129. doi.org: 10.1071/BI9560125

[10] Umetsu K, Kashiwamura S, Suzuki Y. On the identification of the origin of human blood engorged by mosquito 1. Examination of blood groups. Reports of the National Research Institute of Police Science. Research on forensic science.1979; 32(1): 27–32. ISSN: 02857960 [in Japanese]

[11] Umetsu K, Yuuki M, Kashiwamura S, Suzuki Y. On the identification of the origin of human blood engorged by mosquito 2. Examination of serotypes and red cell enzyme types. Reports of the National Research Institute of Police Science. Research on forensic science.1980; 33(4): 206–209. ISSN: 02857960 [in Japanese]

[12] Martínez-de la Puente J, Ruiz S, Soriguer R, Figuerola J. Effect of blood meal digestion and DNA extraction protocol on the success of blood meal source determination in the malaria vector *Anopheles atroparvus*. Malar J. 2013; 12:109 doi: 10.1186/1475-2875-12-109 23517864 10.1186/1475-2875-12-109 PMC3608947

[13] Martínez-de la Puente J, Muñoz J, Capelli G, Montarsi F, Soriguer R, Arnoldi D, Rizzoli A, et al Avian malaria parasites in the last supper: identifying encounters between parasites and the invasive Asian mosquito tiger and native mosquito species in Italy. Malar J. 2015; 14: 32 doi: 10.1186/s12936-015-0571-0 25626918 10.1186/s12936-015-0571-0 PMC4318217

[14] Ngo KA, Kramer LD. Identification of Mosquito Bloodmeals Using Polymerase Chain Reaction with Order-Specific Primers. J Med Entomol. 2003; 40(2): 215–222. 12693851 10.1603/0022-2585-40.2.215

[15] Oshaghi MA, Chavshin AR, Vatandoost H. Analysis of mosquito bloodmeals using RFLP markers. Exp Parasitol. 2006; 114(4): 259–264. doi: 10.1016/j.exppara.2006.04.001 16716302 10.1016/j.exppara.2006.04.001

[16] Esther CS, Barbara S, William AH, John DB, Thomas WS. Laboratory and Field Evaluation of Polymerase Chain Reaction-Based Forensic DNA Profiling for Use in Identification of Human Blood Meal Sources of *Aedes aegypti* (Diptera: Culicidae). J Med Entomol. 2000; 37(4): 492–502. 10916289 10.1603/0022-2585-37.4.492

[17] Mukabana WR, Takken W, Seda P, Killeen GF, Hawley WA, Knols BG. Extent of digestion affects the success of amplifying human DNA from blood meals of *Anopheles gambiae* (Diptera: Culicidae). Bull Entomol Res. 2002; 92(3): 233–239. doi: 10.1079/BER2002164 12088540 10.1079/BER2002164

[18] Goran C, Rajna H, Zvonimir V, Jasenka W. Identification of person and quantification of human DNA recovered from mosquitoes (Culicidae). Forensic Sci Int: Genetics. 2014; 8: 109–112. doi: 10.1016/j.fsigen.2013.07.011 24315597 10.1016/j.fsigen.2013.07.011

[19] QIAGEN. QIAamp® DNA Micro Handbook third ed. Tokyo: QIAGEN Press; 2014.

[20] Watanabe G. DNA extraction from forensic samples by use of QIAamp DNA mini kit. Jpn J Forensic Sci Tec. 2004; 9 (1): 49–58. http://doi.org/10.3408/jasti.9.49 [in Japanese]

[21] Kapa Biosystems. KAPA Human Genomic DNA Quantification and QC Kit Technical Data Sheet. Massachusetts: Kapa Biosystems Press: 2017. Available from: https://www.kapabiosystems.com/product-applications/products/next-generation-sequencing-2/hgdna-quantification/

[22] Applied Biosystems. Applied Biosystems StepOne™ and StepOnePlus™ Real-Time PCR Systems Standard Curve Experiments Getting Started Guide. Foster City: Applied Biosystems Press: 2010. Available from: https://tools.thermofisher.com/content/sfs/manuals/cms_046735.pdf

[23] Van L, Nathalie P, Ezequiel C, Jean C. Improved real-time RT-PCR method for high-throughput measurements using second derivative calculation and double correction. BioTechniques. 2005; 38: 287–293. 15727135 10.2144/05382RR05

[24] Applied Biosystems. AmpFlSTR® Identifiler® Plus PCR Amplification Kit User Guide. Foster City: Applied Biosystems Press: 2015.

